# Growing old with HIV — dealing with co-morbidities

**DOI:** 10.1186/1758-2652-13-S4-O25

**Published:** 2010-11-08

**Authors:** W Powderly

**Affiliations:** 1 UCD School of Medicine and Medical Sciences, Health Sciences Centre, Dublin, Ireland

## 

The prevalence of HIV and AIDS among persons 50 years of age and older continues to increase as a consequence both of improved antiretroviral efficacy extending survival among individuals who contracted the disease earlier in the epidemic and continued primary infection in older individuals. Management of older HIV-infected patients is complicated by the presence of comorbidities that are more common with increasing age, such as diabetes mellitus, cancer, and cardiovascular, renal, hepatic, and bone diseases. Some of these conditions have specific links to HIV infection or with its treatment. Others are consequences of remaining alive to reach an older age. While a determination of the relative contribution of age, HIV status, and antiretroviral exposure to these comorbidities is an important research question, there are also important management issues. In particular, it is important to establish if management of these co-morbidities should differ because of the HIV status of the patient. While these research issues are being resolved, the assessment of comorbidities in older persons should become part of routine care, and at the very least routine age-specific guidelines should be used for screening. Management of older persons with HIV should include baseline evaluation of cardiovascular risk and regular monitoring of fasting lipid and glucose levels, renal function, and markers of bone disease. Furthermore, co-morbidities have an important influence on antiretroviral selection, as avoidance of metabolic and other toxicities or drug-drug interactions is a key issue.

